# An Integrated Regulatory Network Based on Comprehensive Analysis of mRNA Expression, Gene Methylation and Expression of Long Non-coding RNAs (lncRNAs) in Myelodysplastic Syndromes

**DOI:** 10.3389/fonc.2019.00200

**Published:** 2019-03-29

**Authors:** Xiaoli Zhao, Hua Yin, Nianyi Li, Yu Zhu, Wenyi Shen, Sixuan Qian, Guangsheng He, Jianyong Li, Xiaoqin Wang

**Affiliations:** ^1^Key Laboratory of Hematology, Department of Hematology, Collaborative Innovation Center for Cancer Personalized Medicine, The First Affiliated Hospital of Nanjing Medical University, Jiangsu Province Hospital, Nanjing Medical University, Nanjing, China; ^2^Department of Haematology, Huashan Hospital, Fudan University, Shanghai, China

**Keywords:** myelodysplastic syndromes, differentially expressed genes, methylated genes, microRNAs, lncRNAs, regulatory network

## Abstract

Myelodysplastic syndromes (MDS) are a heterogeneous group of disorders characterized by ineffective hematopoiesis, defective differentiation of hematopoietic precursors, and expansion of the abnormal clones. The prevalence of MDS has raised great concerns worldwide, but its pathogenetic mechanisms remain elusive. To provide insights on novel biomarkers for the diagnosis and therapy of MDS, we performed high-throughput genome-wide mRNA expression profiling, DNA methylation analysis, and long non-coding RNAs (lncRNA) analysis on bone marrows from four MDS patients and four age-matched healthy controls. We identified 1,937 differentially expressed genes (DEGs), 515 methylated genes, and 214 lncRNA that showed statistically significant differences. As the most significant module-related DEGs, TCL1A, PTGS2, and MME were revealed to be enriched in regulation of cell differentiation and cell death pathways. In addition, the GeneGo pathway maps identified by top DEGs were shown to converge on cancer, immunoregulation, apoptosis and regulation of actin cytoskeleton, most of which are known contributors in MDS etiology and pathogenesis. Notably, as potential biomarkers for diagnosis of MDS, four specific genes (ABAT, FADD, DAPP1, and SMPD3) were further subjected to detailed pathway analysis. Our integrative analysis on mRNA expression, gene methylation and lncRNAs profiling facilitates further understanding of the pathogenesis of MDS, and may promote the diagnosis and novel therapeutics for this disease.

## Introduction

Myelodysplastic syndromes (MDS) are a group of hemopathies featured with various degrees of ineffective hematopoiesis, and confer the host with inherent risk of progression to acute myeloid leukemia (1–4). Although actual epidemiology are unknown, the incidence of MDS is increasing, attributing to the growing aging population, the use of cytotoxic agents in treating diseases, and environmental carcinogens such as organic solvents (1, 5–7). MDS affect mainly elderly patients. Based on previous literatures, the annual incidence of MDS is ~3.5–10 per 100,000 in the general population, while 12–50 per 100,000 in elderly population (3, 4, 8).

Due to the heterogeneity of MDS, the molecular pathogenesis of MDS remains poorly understood, which has significantly hindered the development of new strategies to improve the therapies (7). Although the molecular characterization of MDS continues to be controversial, various studies demonstrated the clonal involvement of the myeloid lineages. Importantly, the clonal mutation and expansion of abnormal myeloid cells are tightly associated with the dysfunction of pluripotent or multipotent hematopoietic cells, which may establish the MDS phenotype and determine natural disease course. The fundamentally complex genetic and biologic abnormalities in pathogenesis are also demonstrated by the heterogeneity of the clinical and morphologic pictures of MDS (8). Even now, the etiology and pathogenesis of MDS remain inadequately characterized.

Gene expression profiling can identify key deregulated genes and pathways and new prognostic gene signatures in MDS. Recent advances in the molecular pathogenesis of MDS are leading to new biological, clinical, and therapeutic insights. Over the course of the past decade, the application of novel high throughput technologies to the study of MDS has led to the identification of recurrently mutated genes in this disease. For example, microarray-based gene expression profiling has expedited the identification of many differentially expressed genes in MDS, and also underscored several critical gene pathways deregulated in this hematopoietic malignancy (9). Comprehensive genomic profiling of MDS and acute myeloid leukemia (AML) cases have remarkably enabled the detection of genes functioning as drivers of differentiation and subclonal mutations, which could profoundly facilitate the timely diagnosis, accurate risk prognostication and targeted therapies (10).

The aim of this study is to construct an integrated, MDS-specific regulatory network, further providing novel MDS signature genes, and pathways that can be translated to potential novel biomarkers or approaches for the diagnosis and therapy of MDS. We performed high-throughput genome-wide mRNA expression profiling, DNA methylation analysis, and lncRNA analysis on bone marrows from four MDS patients and four age-matched healthy controls. Genomic profiling was used for construction of an integrated regulatory network. Additionally, GO analysis and pathway enrichment analysis for MDS were performed. Notably, four MDS-specific genes identified as potential biomarkers in diagnosis of MDS were analyzed in detail by pathway analysis. Our findings provide complementary understanding of MDS pathogenesis, and lead to a better insight into the development of novel strategies for diagnosis and therapy of this disease.

## Materials and Methods

### Patients and Samples

A cohort of four MDS patients and four healthy controls were included in this integrative parallel study on high-throughput global gene profiling, as previously reported (11). The subtype of four MDS patients was refractory anemia with excess blasts (RAEB), including three male and one female, and the median age was 67 years. Diagnosis of the MDS patients was based on the criteria defined by the International System for Human Cytogenetic Nomenclature. Bone marrow (BM) mononuclear cells samples were isolated by Ficoll solution (GE Healthcare) according to the manufacturer's instructions. Ethical clearance and approval was obtained from the local institutional research board (Code, 2009M-012). The study protocol was carefully explained to the participants and participation was fully voluntary. Written informed consent was obtained from all participants and they agreed to publish their individual data. All procedures were done according to the standard with a minimum risks.

### DNA Extraction and Microarray-Based Genomic Profiling

Genomic DNA was isolated using a QIAamp DNA Blood Mini Kit (Qiagen) from BM samples according to the standard procedures. The Infinium BeadChip DNA methylation array was carried out for profiling of human whole genome genes, includes CpG sites, CpG islands and non-coding RNA, as previously reported (11). A total of 27,958 Entrez Gene RNAs and 7,419 lincRNAs were included in the 60K BeadChips for human genome-wide gene expression (Agilent). Original data have been deposited in Gene Expression Omnibus (GEO) of the National Center for Biotechnology Information, with public online access links (GSE51758 and GSE51757).

### Differentially Expressed Genes (DEGs) Screening

R software was used to process the microarray data (12). Limma (Linear Model for Microarray) package in R was used to identify DEGs between MDS patients and control group (13). After the data was normalized by log2 transformation, the Bayes moderated *t*-test (4) was recruited for the identification of DEGs. The screening criterion for DEGs was set up as the FDR (false discovery rate) <0.05 and |log FC (fold change)| >1.

### Construction of Integrated Regulatory Network

Systematic integration of various high-throughput datasets was performed for the construction of integrated regulatory network. Targets of one microRNA (miRNA) with strong experimental evidence were obtained in the mirTARbase and DIANA-TarBase v8 as known targets. TargetScan version 6.2, miRBase version 18, Diana version 4.0 and targetMiner were used to aggregate potential targets sets. DEGs predicted by more than three tools were selected. The Cytoscape software (http://cytoscape.org/) was employed for the final assembly of the integrated gene regulatory network.

### GO and KEGG Enrichment Analyses of DEGs

DAVID (https://david.ncifcrf.gov/) was employed to perform Gene Ontology (GO) enrichment analysis, based on the hypergeometric distribution method. The criteria of count number ≥2 and *P*-value < 0.05 were selected as the threshold for GO enrichment. Kyoto Encyclopedia of Genes and Genomes (KEGG) pathway enrichment analysis was performed, as previously described (https://www.genome.jp/kegg/), to identify the main functional pathways in DEGs involved in metabolism of MDS. The most significantly enriched pathway-related DEGs were mapped to the corresponding KEGG pathways. The thresholds were set as count number <3 and *P*-value < 0.05.

## Results

### A Network Framework of Data Integration

**Figure 1:**
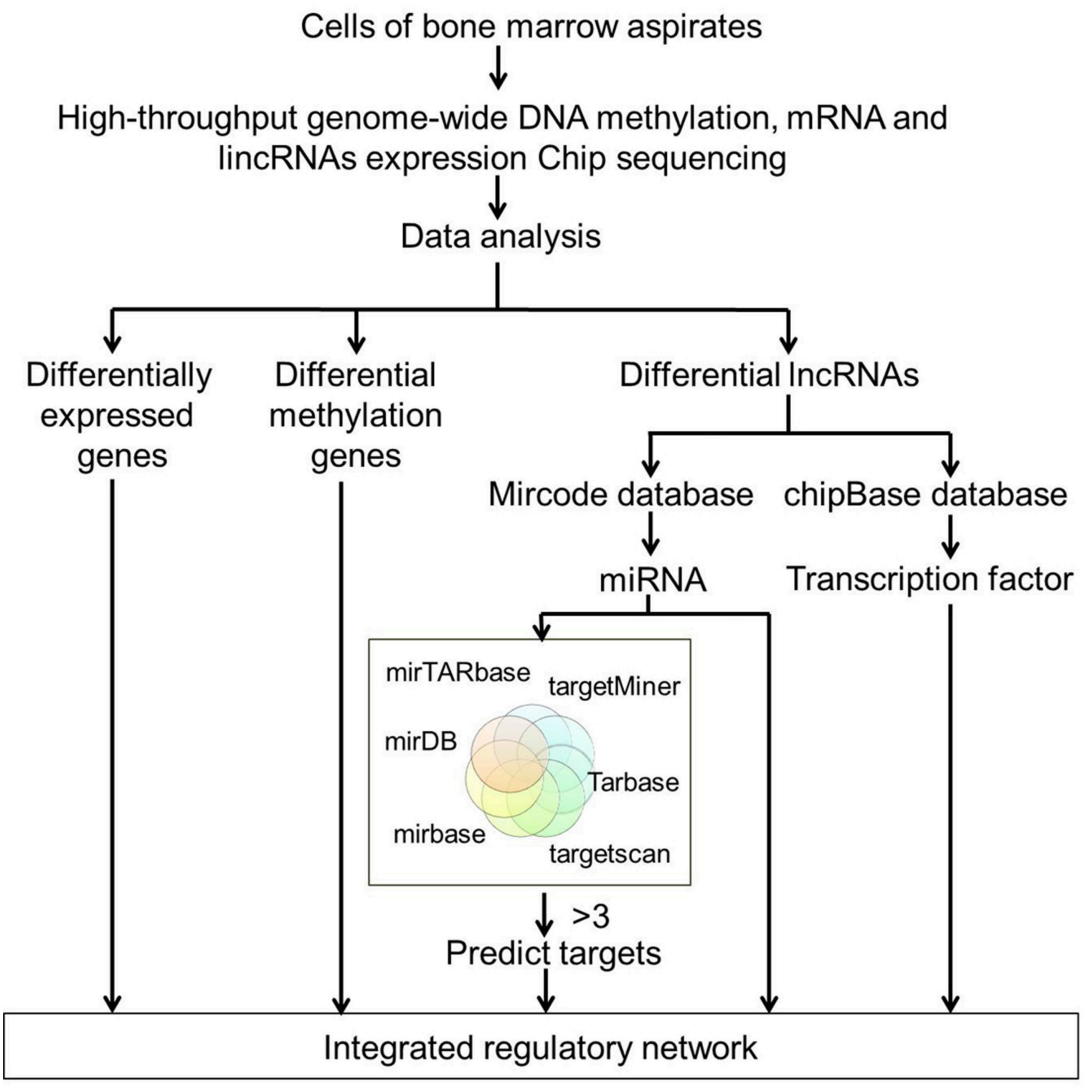
Flowchart of high-throughput analysis for data integration. High-throughput genome-wide DNA methylation, mRNA, and lncRNAs expression profiling were performed on BM samples from four MDS patients and four age-matched healthy controls. DEGs, differentially methylated genes and differentially expressed lncRNAs were initially screened out. miRNAs were identified using regulatory data collected from the microcode database. Target genes were predicted by six databases including the mirTARbase, Tarbase, TargetScan version 6.2, miRBase 18, Diana version 4.0, and targetMiner. All the DEGs, differentially methylated genes, and differentially expressed lncRNAs-predicted targets were integrated to construct the regulatory network of MDS.

**Figure 2:**
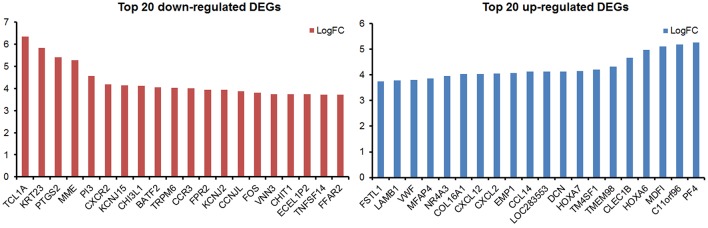
The top 20 up-regulated and top 20 down-regulated DEGs in this study.

By analyzing the microarray data from four MDS and four control samples according to designed flowchart (Figure 1), we identified 1,937 DEGs, 515 differentially methylated genes and 214 differentially expressed lncRNAs. These 1,937 DEGs included 853 up-regulated DEGs and 1,084 down-regulated DEGs. The top 20 up-regulated and top 20 down-regulated DEGs are shown in Figure 2. Among these top deregulated genes, while three up-regulated DEGs (CCL14/CXCL2/PF4) and one down-regulated DEG (TNFSF14) are involved in immune response, one down-regulated DEG (CXCR2) is associated with apoptosis. The rest of the genes are involved in cell differentiation, regulation of biosynthetic process, regulation of intracellular protein transport, protein amino acid phosphorylation and so on. Of these 1,937 DEGs, 1,606 DEGs have their own names, whereas 1,592 DEGs can be identified in the DAVID database. Besides, 28 of 214 lncRNAs have the record of transcripts. Using regulatory data collected from the microcode database, we identified 72 microRNAs. Subsequently, 1,348 genes were predicted as potential targets of these 72 mircoRNA through six databases: mirTARbase, Tarbase, TargetScan version 6.2, miRBase 18, Diana version 4.0, and targetMiner. Then we focused on these genes for further analysis to construct an integrated MDS regulatory network.

### Construction of Global Gene Profiling Based Integrated Regulatory Network for MDS

**Figure 3:**
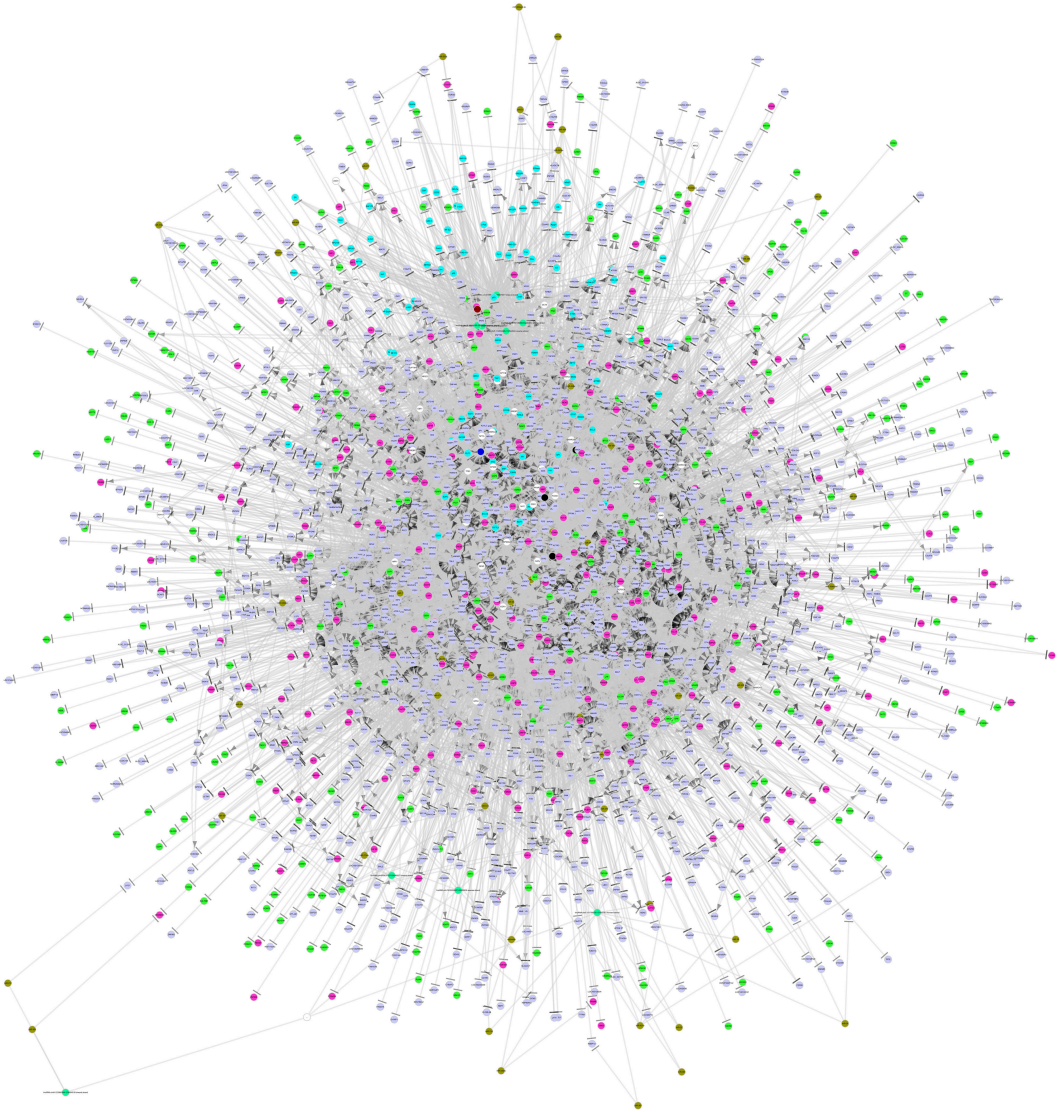
The integrated regulatory network of genes in the pathogenesis of MDS. This gene network included three major network components: TF-gene-lncRNA regulatory network, TF-miRNA-lncRNA regulatory network, and miRNA-gene-methylation regulatory network. TF, transcription factor; miRNA, microRNA; lncRNA, long non-coding RNA.

By integrating a large number of hypermethylated genes, DEGs, DEG-related miRNAs, transcription factors (TFs) and lncRNAs collected from the above-mentioned six databases, a regulatory network was built. As shown in Figure 3, this network included three major network components: TF-gene-lncRNA regulatory network, TF-miRNA-lncRNA regulatory network and miRNA-gene-methylation regulatory network.

### Identification of MDS Related Functions and Pathways

#### GO Analysis of Module-Related DEGs

**Figure 4:**
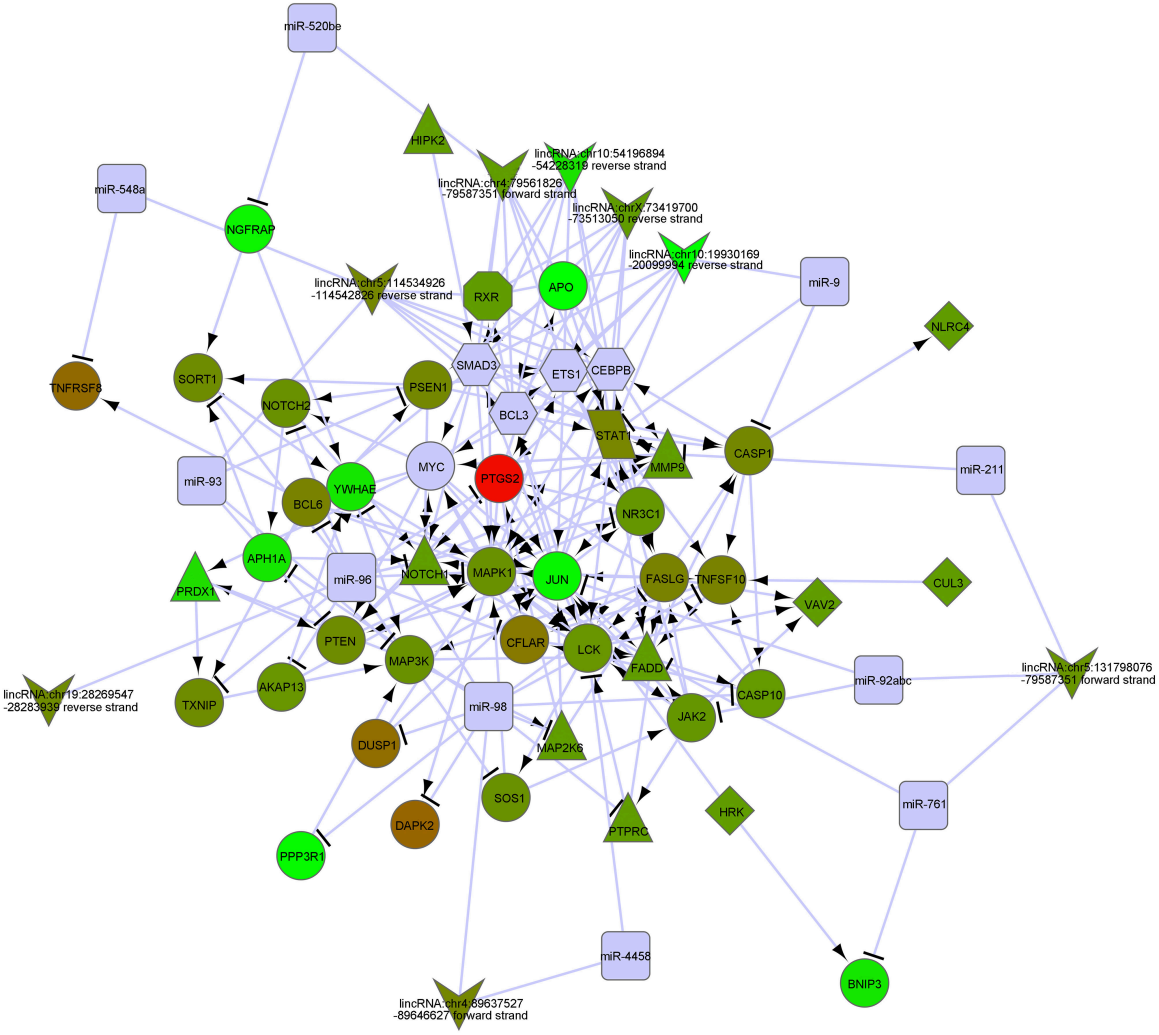
The gene networks in regulation of apoptosis by GO analysis of modulate-related DEGs.

In order to biologically evaluate the MDS-specific gene regulatory network, we carried out GO analysis. Specifically, we evaluated our network based on functional knowledge about genes that are involved in similar biological processes. We found that all biological processes are internally connected. While the number for maximally overlapped DEGs of two biological processes was 130, the number for minimally overlapped DEGs of two biological processes was one. To gain further insight into the function of DEGs in our network modules, module-related DEGs were annotated by the online biological classification software DAVID. Some target genes are statistically enriched in the GO terms of apoptosis and immune response. As shown in Table 1, the top ten enriched GO terms include: intracellular signaling cascade, regulation of apoptosis (Figure 4), phosphate metabolic process, immune response, defense response, regulation of cell proliferation, positive regulation of biosynthetic process, phosphorylation, regulation of transcription from RNA polymerase II promoter, and biological adhesion.

**Table 1:** The top 10 enriched GO terms revealed by GO analysis of module-related DEGs in this study.

**Biology process**	**Count**	**%**	***P*-value**
Intracellular signaling cascade	160	8.040201	0.003180933
Regulation of apoptosis	127	6.38191	8.46576E-07
Phosphate metabolic process	123	6.180905	0.012612388
Immune response	122	6.130653	2.39405E-09
Defense response	102	5.125628	1.34779E-06
Regulation of cell proliferation	102	5.125628	0.01192571
Positive regulation of biosynthetic process	99	4.974874	0.000806854
Phosphorylation	99	4.974874	0.040864467
Regulation of transcription from RNA polymerase II promoter	97	4.874372	0.006699243
Biological adhesion	93	4.673367	0.009089611

#### Pathway Analysis of Module-Related DEGs

**Figure 5:**
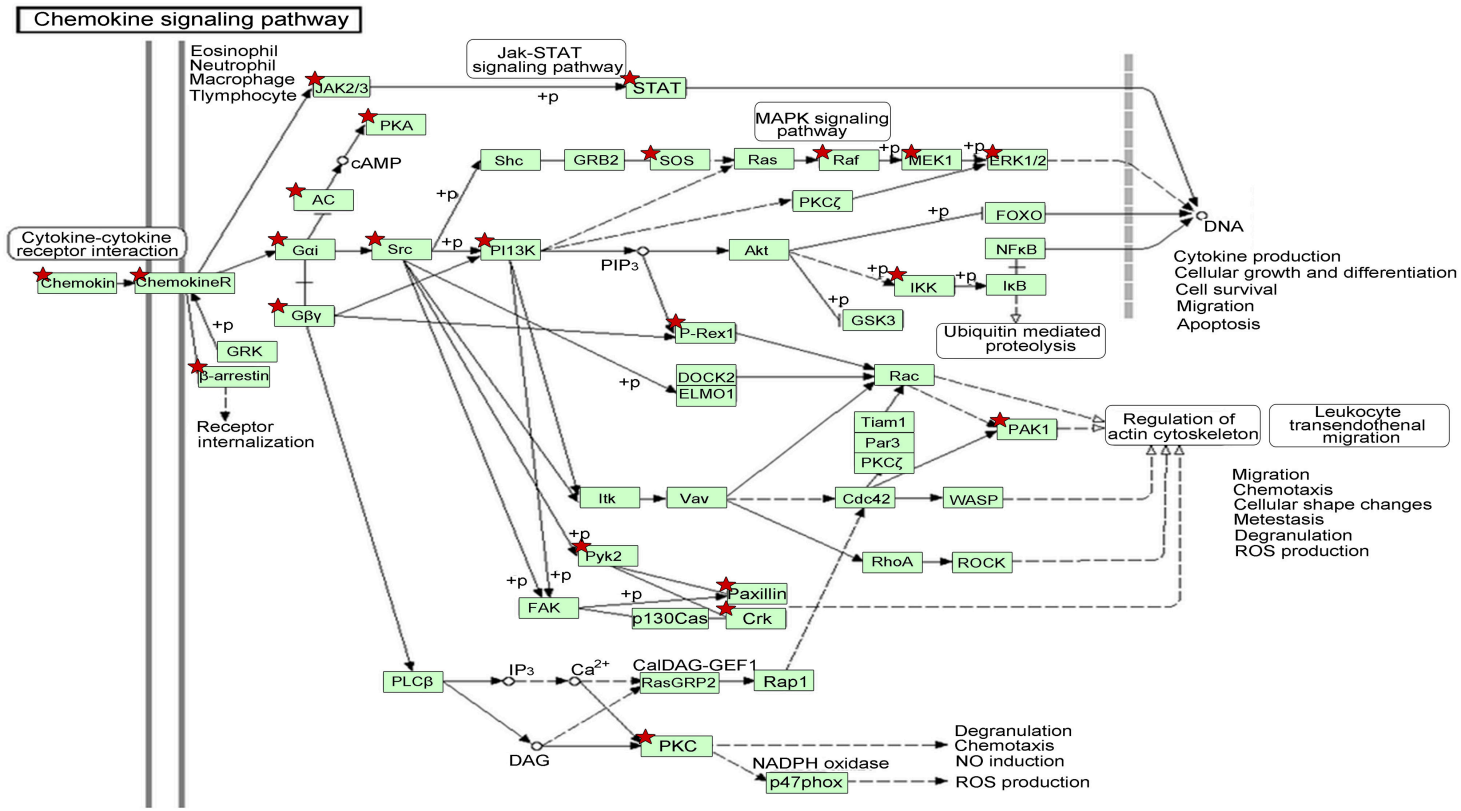
The gene network on chemokine signaling pathway by KEGG analysis of module-related DEGs. The asterisk indicates the significant DEGs involved in this pathway.

We also identified the KEGG pathways that are significantly enriched in MDS patients, and many of these pathways are associated with cancer. The top ten enriched KEGG pathways include: pathways in cancer, MAPK signaling pathway, chemokine signaling pathway (Figure 5), regulation of actin cytoskeleton, focal adhesion, endocytosis, leukocyte transendothelial migration, natural killer cell mediated cytotoxicity, neurotrophin signaling pathway, and insulin signaling pathway (Table 2). The top GeneGo pathway maps regulated by DEGs converged on immunoregulation, apoptosis and cell adhesion, most of which are known to be contributors in MDS etiology and pathogenesis.

**Table 2:** The top 10 enriched KEGG pathways revealed by pathway analysis of module-related DEGs in this study.

**ID**	**Term**	***P*-value**	**Count**	**%**
hsa05200	Pathways in cancer	0.001124	55	2.763819
hsa04010	MAPK signaling pathway	0.014493	42	2.110553
hsa04062	Chemokine signaling pathway	7.46E-06	42	2.110553
hsa04810	Regulation of actin cytoskeleton	0.009778	36	1.809045
hsa04510	Focal adhesion	0.003298	36	1.809045
hsa04144	Endocytosis	0.067678	28	1.407035
hsa04670	Leukocyte transendothelial migration	0.001809	25	1.256281
hsa04650	Natural killer cell mediated cytotoxicity	0.01692	24	1.20603
hsa04722	Neurotrophin signaling pathway	0.007409	24	1.20603
hsa04910	Insulin signaling pathway	0.097467	21	1.055276

In order to better understand the mechanisms underlying the interactions between the pathway-related DEGs, we mapped the DEGs in the above-mentioned pathways to their corresponding KEGG pathways (Figure 5). As shown in the map, for example, the Raf-1 proto-oncogene RAF1 was involved in more than 20 pathways, including apoptosis and regulation of cell migration. The genes STAT and PAK1 were simultaneously involved in more than two pathways.

#### In-depth Pathway Analysis of Four Candidate DEGs for MDS Diagnosis

Our previous work has demonstrated that there were six DEGs identified as potential biomarkers for diagnosis of MDS, including ABAT, DAPP1, FADD, LRRFIP1, PLBD1, and SMPD3 (11). In order to better verify the potential diagnostic value and confirm their values as potential therapeutic targets, we performed a detailed in-depth pathways analysis for these six DEGs. However, as shown in Table 3, only four genes (ABAT, DAPP1, FADD, and SMPD3) were identified to possess their functional pathways.

**Table 3:** The functional pathways of four MDS-specific genes with potential values in diagnosis and therapeutically targeting.

**Gene symbol**	**Gene name**	**Pathway**
ABAT	4-aminobutyrate aminotransferase	hsa00250: alanine, aspartate, and glutamate metabolism
		hsa00280: valine, leucine, and isoleucine degradation
		hsa00410: beta-alanine metabolism
		hsa00640: propanoate metabolism
		hsa00650: butanoate metabolism
FADD	Fas (TNFRSF6)-associated via death domain	hsa04210: apoptosis
		hsa04620: toll-like receptor signaling pathway
		hsa04622: RIG-I-like receptor signaling pathway
		hsa05200: pathways in cancer
		hsa05010: Alzheimer's disease
DAPP1	Dual adaptor of phosphotyrosine and 3-phosphoinositides	hsa04662: B cell receptor signaling pathway
SMPD3	Sphingomyelin phosphodiesterase 3	hsa00600: sphingolipid metabolism

## Discussion

MDS are a heterogeneous group of hematopoietic neoplasms characterized by defective differentiation of hematopoietic stem cells and bone marrow dysplasia (1). These characteristics limit the deeper going into its etiology and pathogenesis. The outcomes of MDS patients have not substantially improved over the last few decades, due to the lack of a comprehensive understanding of the pathogenesis. In this study, we completed the construction of an integrated MDS regulatory network, which not only facilitates the further understanding of the pathogenesis of MDS, but also can promote the diagnosis and novel therapeutics for this disease.

Currently, the application of new high-throughput technologies is promising to break the frontiers in the field of MDS etiology and pathogenesis. It has been proved that the network analysis is useful in unraveling the complexity of biological regulations (14–22). For instance, aggressive behavior of breast cancer is related to cell proliferation and hormone stimulus via an integrated gene regulatory network (23). Cancers share a common regulatory network involving histones and regulators of the cell cycle and immune responses by mining gene expression data, which have been demonstrated by Knaack et al. (24). Lots of mutated genes and driver pathways are important for the pathogenesis of MDS using RNA-seq technology (7). In addition, Tawana et al. believe that we will have a better understanding of the role of an increasing number of inherited genetic factors on MDS/AML incidence and management through high-throughput gene exploring (10).

Our global genome profiling between MDS patients and healthy controls resulted in more down-regulated DEGs (1034/1937) than up-regulated DEGs (853/1937). The majority of up-regulated DEGs were correlated to immune responses (total 122 DEGs). Meanwhile, down-regulated DEGs were mainly enriched in regulation of cell death. For example, the most significantly down-regulated, module-related DEGs (TCL1A, PTGS2, and MME) were revealed to be enriched in regulation of the cell differentiation and cell death pathways. In this study, the top GeneGo pathway maps regulated by DEG converge on cancer, immunoregulation, apoptosis, cell adhesion, and regulation of actin cytoskeleton, most of which contribute significantly to the oncogenesis of MDS. These pathways in cancer are likely correspondent to the pre-leukemic status of MDS. For instance, cell adhesion is proved as cancer metastasis-related pathways (16), and others may contribute to the hallmarks of morphologic dysplasia observed in MDS, due to apoptosis, regulation of the cytoskeleton and immunoregulation (7).

LncRNAs have been involved in the regulation of a variety of biological functions in both physiological and pathogenic conditions. Many researchers have detected aberrantly expressed lncRNAs in human cancers (18, 25–30) and found that these lncRNAs are implicated in the regulation of many critical oncogenes or tumor suppressor genes, such as p53 (31). By targeting other genetic and epigenetic regulators, lncRNAs can also regulate the transcriptional and translational output in cells (32). Many studies have reported that lncRNAs have been identified as important participants in the pathogenesis and disease progression of AML. So it seems possible that profiling of their expression would have significant impact on MDS diagnosis and prognosis. Yao et al. provide evidence that higher expression levels of four lncRNAs in their scoring system are associated with distinct clinical and genetic features, and they conclude that the high abundance of these lncRNAs could predict inferior outcomes of MDS patients (33). Moreover, cancer associated lncRNAs may serve as diagnostic or predictive biomarkers, and also imply new therapeutic strategies focusing on selectively silencing these lncRNAs in treating cancers (34). In this study, we identified 214 lncRNAs, and 28 of them had the record of transcripts. However, further in-depth studies are still required to thoroughly reveal the functions of these lncRNAs and their significances in MDS.

Epigenetic alterations is widely accepted as a common event in carcinogenesis, and aberration of epigenetic regulation may be considered functionally equivalent to classical genetic alterations (11). DNA methylation has an important role in hematological malignancies, and accumulating data indicate that epigenetic modifications are attractive therapeutic targets in any oncopathological state (35). We identified 515 methylated MDS-specific genes in our investigation on global gene profiling. In our previous work, we defined six genes (ABAT, DAPP1, FADD, LRRFIP1, PLBD1, and SMPD3) as CIMP (the CpG island methylator phenotype) markers of MDS, which suggests a non-invasive approach for the diagnosis and prognosis of MDS (11). In this study, we further analyzed the data to pinpoint miRNA, lncRNAs associated with these six genes. Four genes (ABAT, DAPP1, FADD, and SMPD3) were identified to possess their functional pathways. Further studies will be carried out by our research group to unveil their significances in pathogenesis of MDS. Through using high-throughput technologies in this era, the incorporation of more relevant parameters would undoubtedly move us one step further toward the diagnosis and therapy of MDS.

## Conclusion

In summary, we performed microarray on BM samples from four MDS patients and four age-matched healthy controls. In order to construct an integrated regulatory network to uncover potential novel biomarkers for the diagnosis and therapy of MDS, we undertook multiple analyses, including high-throughput genome-wide mRNA expression profiling, DNA methylation analysis, and lncRNAs analysis. Additionally, GO analysis and pathway enrichment analysis for MDS were performed. Meanwhile, six MDS-specific genes previously identified as potential biomarkers in MDS diagnosis were re-analyzed in detail. Our findings provide valuable resources for in-depth understanding the pathogenesis of MDS, and also lead to better insights on developing novel strategies for diagnosis and therapy of this disease.

## Author Contributions

JL and XW designed the experiments. XZ and HY carried out most of the experiments and analyzed the data. NL, YZ, WS, SQ, and GH assisted in analyzing the data. XZ wrote the article.

### Conflict of Interest Statement

The authors declare that the research was conducted in the absence of any commercial or financial relationships that could be construed as a potential conflict of interest.
